# Plasma Biomarkers for Accelerated Cognitive Decline: A Tool for Targeted Clinical Trial Recruitment

**DOI:** 10.1002/alz.086334

**Published:** 2025-01-09

**Authors:** Federica Anastasi, Armand González Escalante, Pol Segura‐Retana, Paula Ortiz‐Romero, David López‐Martos, Gonzalo Sánchez‐Benavides, Carolina Minguillon, Marta del Campo, Marc Suarez‐Calvet

**Affiliations:** ^1^ Centro de Investigación Biomédica en Red de Fragilidad y Envejecimiento Saludable (CIBERFES), Madrid Spain; ^2^ Barcelonaβeta Brain Research Center (BBRC), Pasqual Maragall Foundation, Barcelona Spain; ^3^ IMIM (Hospital del Mar Medical Research Institute), Barcelona Spain; ^4^ Centre for Genomic Regulation (CRG), Barcelona Institute of Science and Technology (BIST), Barcelona Spain; ^5^ Universitat Pompeu Fabra, Barcelona Spain; ^6^ Universitat Politècnica de Catalunya (UPC), Barcelona Spain; ^7^ Hospital del Mar Research Institute (IMIM), Barcelona Spain; ^8^ Centro de Investigación Biomédica en Red de Fragilidad y Envejecimiento Saludable (CIBERFES), Instituto de Salud Carlos III, Madrid Spain; ^9^ Servei de Neurologia, Hospital del Mar, Barcelona Spain

## Abstract

**Background:**

Detecting Alzheimer's disease (AD) biological hallmarks before clinical symptoms emerge is now possible with available blood‐based biomarkers. However, the rate of cognitive decline varies among individuals at risk of AD, and accurate prognostic blood‐based biomarkers are lacking. Our goal is to identify plasma proteins predictive of fast cognitive decline in asymptomatic individuals at risk of AD.

**Method:**

We studied 93 cognitively unimpaired individuals from the ALFA+ cohort with two longitudinal cognitive assessments (3‐year follow‐up, Preclinical Alzheimer Cognitive Composite, PACC). PACC‐changes were calculated as the change in PACC scores between two visits divided by the time elapsed. Adjustments for demographic variables associated with PACC‐change (age and education years) were made by residualizing. Two groups were defined based on residual median values, namely the slow‐ and the fast‐cognitive decliners groups. Protein levels in plasma were measured using Proximity Extension Assay (PEA) technology (Olink).

**Result:**

In a preliminary analysis, non‐parametric univariate analysis of baseline proteomics data identified 74 significantly different proteins between slow‐ and fast‐cognitive decliners. Specifically, 29 proteins were upregulated in fast decliners, and 45 in slow decliners, with the latter enriched in REACTOME Pathways of Cytokine Signalling and Immune System. Synaptic proteins were among the most significantly deregulated.

**Conclusion:**

Plasma protein signatures at baseline differ between individuals with fast and slow rates of cognitive decline. Early identification of those with rapid disease progression is crucial for efficient clinical trial recruitment. Faster cognitive decline can shorten testing periods, enhancing trial efficiency and expediting the assessment of potential interventions.

